# Regulation of *Candida albicans* Hyphal Morphogenesis by Endogenous Signals

**DOI:** 10.3390/jof5010021

**Published:** 2019-02-28

**Authors:** Daniel Kornitzer

**Affiliations:** Department of Molecular Microbiology, B. Rappaport Faculty of Medicine, Technion-Israel Institute of Technology, Haifa, Israel; danielk@technion.ac.il

**Keywords:** *Candida albicans*, hyphae, morphogenesis, cell cycle, transcription

## Abstract

*Candida albicans* is a human commensal fungus that is able to assume several morphologies, including yeast, hyphal, and pseudohyphal. Under a range of conditions, *C. albicans* performs a regulated switch to the filamentous morphology, characterized by the emergence of a germ tube from the yeast cell, followed by a mold-like growth of branching hyphae. This transition from yeast to hyphal growth has attracted particular attention, as it has been linked to the virulence of *C. albicans* as an opportunistic human pathogen. Signal transduction pathways that mediate the induction of the hyphal transcription program upon the imposition of external stimuli have been extensively investigated. However, the hyphal morphogenesis transcription program can also be induced by internal cellular signals, such as inhibition of cell cycle progression, and conversely, the inhibition of hyphal extension can repress hyphal-specific gene expression, suggesting that endogenous cellular signals are able to modulate hyphal gene expression as well. Here we review recent developments in the regulation of the hyphal morphogenesis of *C. albicans*, with emphasis on endogenous morphogenetic signals.

## 1. Introduction

*Candida albicans* is a human commensal organism found in the gastrointestinal tract and other mucosal surfaces of a majority of the population [1,2,3]. It can cause superficial mucosal infections in immunocompetent individuals, but among immunocompromised or debilitated patients, it can be responsible for life-threatening systemic disease [4]. In the United States, 9% of nosocomial bloodstream infections are caused by *Candida* spp. [5,6], of which 40%–70% are caused by *C. albicans*, and the rest by other *Candida* species [7,8]. Bloodstream infection, or candidemia, develops into deep-seated candidiasis when the fungus invades internal organs [9]. The mortality rate for invasive candidiasis has remained stubbornly high at 30%–40% over the last several decades in spite of the introduction of new classes of antifungals such as the echinocandins [6,8,10,11,12,13,14]. Candidiasis is currently responsible for an estimated 700,000 annual deaths worldwide [15].

*C. albicans* has been historically distinguished from other *Candida* species by its ability to switch between a yeast form of growth, with rounded cells that disperse after septation; a pseudohyphal form, characterized by chains of elongated yeast cells; and a hyphal, or mold form, characterized by branching chains of tubular cells without constrictions at the sites of septation [16]. While most other *Candida* species are only able to form yeast and pseudohyphae (reviewed in Reference [17]), *C. dubliniensis* and *C. tropicalis*, the *Candida* species most closely related to *C. albicans,* are nonetheless also able to form true hyphae, albeit less efficiently and under a more restricted range of conditions [18,19]. Recently, the emerging pathogen *C. auris* was found to form hyphae as well, but only after passage in an animal host [20].

Several environmental stimuli can promote the switch from yeast to hyphal growth in *C. albicans*. Incubation in serum at 37 °C is a potent stimulus and provides the basis for a diagnostic test for *C. albicans* in the clinical laboratory. Other environmental stimuli that are known to promote the switch to hyphal growth in *C. albicans* include neutral or alkaline pH, carbon starvation, nitrogen starvation, cell density via quorum sensing molecules, low oxygen and elevated CO_2_, and the presence of *N*-acetylglucosamine (GlcNAc) (reviewed in References [21,22]). 

## 2. The Role of Hyphal Morphogenesis in the Virulence of *C. albicans*

The observation that *C. albicans* is found predominantly in the hyphal form in tissue samples of candidiasis patients [23] suggests that the yeast-to-hyphal morphogenetic switch plays a role in the transition from candidemia to the subsequent tissue invasion. The establishment of candidemia itself might also be aided by the enhanced ability of the hyphal morphology to penetrate the mucous membranes and underlying tissues, and to enter the bloodstream [24]. Furthermore, the hyphae formation in the phagosome was shown to contribute to the ability of *C. albicans* cells to escape phagocytosis and kill the macrophage [25,26,27]. Finally, optimal biofilm formation on synthetic substrates, an ability that enables *C. albicans* to colonize indwelling devices and cause iatrogenic candidemia, is highly correlated with the capacity of the strain to form hyphae [28]. Animal models of infection provide support for the role of the yeast to hyphal transition in pathogenesis. In a mouse model of systemic infection, *C. albicans* mutants that are unable to switch from the yeast form to the hyphal form demonstrate significantly reduced virulence [29,30]. Studies of strains engineered so that the yeast-to-hyphal switch can be regulated in vivo suggested that hyphal morphogenesis after injection into the bloodstream is essential for virulence [29,31] while inhibiting hyphal morphogenesis early in the infection significantly increased the survival of the host [32]. Whereas a genomic screen to identify determinants of hyphal growth and/or virulence in mice revealed only a partial overlap [33], a more recent global analysis reaffirmed the link between hyphal morphogenesis and virulence by showing that among 177 mutant strains tested for virulence in mice, attenuation of virulence was significantly correlated with decreased hyphal morphogenesis [34]. On the other hand, most studies and observations appear to support a strong link between hyphal morphogenesis and *C. albicans* pathogenicity [35,36]. 

Mucosal infections that affect both healthy and immunosuppressed individuals include oral candidiasis and vulvovaginal candidiasis, whereas esophageal candidiasis occurs in patients with chronic diseases [37]. These types are much more prevalent than systemic candidiasis and, although usually less threatening than invasive disease, can impose a significant burden on patients [38]. These superficial infections appear to involve the yeast-to-hyphal transition as well [39,40,41,42].

## 3. Mechanism of Hyphal Morphogenesis

The two main fungal morphologies, yeast and hyphae, are distinguished by a different polarization of cellular growth. In both cases, the growth is differentially distributed: in hyphae, the growth is concentrated at the tip of the extending filament, and in yeast, the growth occurs mainly in the bud and the daughter cell, and very little in the mother cell. However, in contrast to hyphal growth which occurs permanently at the apex, the yeast bud only maintains apical growth in the initial stages after emergence from the mother cell, after which it switches to isotropic growth, lending the cell its final oval morphology. The transition from apical to isotropic growth in the yeast bud is best understood in the baker’s yeast *S. cerevisiae*, where it depends on the sequential activation of the different cyclin–cyclin-dependent kinase (CDK) complexes involved in the cell cycle progression [43]. In the early bud, the activity of the G1 cyclins Cln1 and Cln2 promotes focused apical growth by concentrating the activity of the small GTPase Cdc42 at the bud tip, which induces a polarization of the actin cytoskeleton towards the tip, whereas in G2, activation of the mitotic cyclins causes a switch from apical to diffuse isotropic growth and the delocalization of Cdc42 from the bud tip (reviewed in Reference [44]). It was, therefore, suggested that the difference between the yeast and hyphal modes of growth in polymorphic fungi such as *C. albicans* can be reduced largely to a difference in the polarization of the actin cytoskeleton [45]. 

In filamentous fungi, the localization of cellular growth into a small area of the cell surface at the hyphal apex requires strong polarization of the cellular biosynthetic machinery, involving a large-scale movement of vesicles containing membranes and cell wall precursors towards the hyphal tip (recently reviewed in References [46,47]). This movement depends on both the microtubule and the actin cytoskeleton, and is coordinated by a vesicle organizing center located just behind the hyphal tip: the Spitzenkörper. The rapid exocytosis of the transported vesicles drives hyphal tip elongation. This exocytosis must be counterbalanced by endocytosis in order to recuperate excess membranes and membrane-anchored enzymes that participate in cell wall biosynthesis [48,49]. A collar of endocytic actin patches surrounds the hypha subapically, suggesting a tight coupling between apical exocytosis and subapical endocytosis [50]. Although the mechanics of hyphal elongation are thought to be broadly similar in *C. albicans* hyphal cells and in filamentous fungi, there are, nonetheless, some significant differences: for example, the *C. albicans* hyphae extension is relatively slower and does not appear to require microtubules, although it does still require the actin cytoskeleton [51,52]. Furthermore, while *C. albicans* hyphae also have a Spitzenkörper near the apex [53], a recent report concludes that the travel of most secretory vesicles is short-range, compared to that in typical filamentous fungi [54]. 

The establishment and maintenance of polarization of the hyphal cytoskeleton require polarity markers at the hyphal apex. As in other fungi, a protein complex called polarisome forms a cap at the site of growth in *C. albicans* hyphae, as well as in yeast and pseudohyphal cells [53]. Compared to the Spitzenkörper, the hyphal polarisome proteins show much less turnover [55]. The polarisome recruits the formin Bni1, which, in turn, may stimulate actin polymerization at the hyphal tip [56,57]. Polarized growth in *C. albicans*, like in many cell types, also requires several small GTPases such as Cdc42 [58,59] and other Ras-like proteins (reviewed in Reference [60]). The regulation of Cdc42 activity and localization by the activity of its GTP exchange factor Cdc24 and by its GTPase-activating proteins Rga2 and Bem3 are thought to be central to the regulation of hyphal morphogenesis [61,62]. Another small GTPase, Rsr1, is also required for the maintenance of the polar localization of Cdc42 [63]. In particular, Rsr1 is required for the contact-dependent (thigmotropic) directionality of hyphal growth: in its absence, hyphae lose their ability to follow the contour of solid growth surfaces [64,65].

## 4. Induction of the Hyphal Morphogenesis of *C. albicans* by External Stimuli

Several signal transduction regulators, notably components of the MAPK- [66] and cAMP/PKA-dependent pathways [67,68], can mediate the yeast-to-hyphal switch. Hyphal morphogenesis is accompanied by the increased expression of a large number of genes [69,70], and many transcription factors (TFs) have been identified that can influence filamentous growth, including Cph1 [71], Efg1 [30,72], Cph2 [73], Czf1 [74], Tec1 [75], Rim101 [76], Hms1 [77], Tup1 [78], Nrg1 [79], Flo8 [80], Brg1/Gat2 [81], Mcm1 [82], Fkh2 [83] and Ume6 [84,85]. Deletions of the genes encoding these TFs exhibited reduced filamentous growth (or, in the case of the transcriptional repressors Nrg1 and Tup1, enhanced filamentous growth). While mutants of some TFs such as Efg1 are defective in hyphal morphogenesis induced by many conditions, the effect of the deletion of many of these TFs is only detectable under specific induction conditions. This suggests that specific TFs can be activated by specific signaling pathways and, indeed, several of these TFs are direct targets of hyphal-inducing signal transduction pathways [66,86,87]. The transcription program that accompanies the yeast-to-hyphal switch also varies according to the mode of hyphal induction [69,70,88]. However, a “core” set of induced genes common to many hyphal induction modes can be defined, which includes genes encoding hyphal cell surface components such as the Hwp1 and Als3 proteins, and the cytolytic toxin Ece1 [89]. 

Although most of the transcription factors above are necessary for hyphal growth at least under some conditions, their ectopic expression is not sufficient to induce authentic hyphal morphogenesis or the full hyphal gene expression profile. One exception is Ume6, high artificial expression of which can induce hyphae whilst intermediate expression causes pseudohyphal chain formation [85,90]. Conversely, *ume6^−/−^* mutants, while capable of initiating germ tube formation when transferred to hyphal-inducing conditions, are profoundly defective in hyphal extension under most tested conditions [84,85]. The *UME6* gene is regulated by many of the transcription factors required for hyphal morphogenesis, including Cph1, Tec1, Flo8, Rfg1 and Nrg1 [84,85].

**Figure 1:**
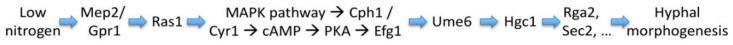
A simplified outline of one *C. albicans* hyphal morphogenesis signaling pathway.

The environmental inputs that activate the hyphal growth pathways are varied, as mentioned above, and can activate distinct signal transduction pathways. Among the best characterized are the signal transduction pathways that involve the ammonium permease Mep2 [91] and the G-coupled receptor Gpr1 [92]. On the one hand, via Ras (in part at least), these activate both the AMP cyclase Cyr1/Cdc35 and the protein kinase A pathway, thus activating the transcription factor Efg1 [86,93], and, on the other hand, they activate Cph1 via the MAP kinase pathway [94,95]. The temperature signal was shown to depend on the chaperone Hsp90 and the transcription factor Hms1, as well as the CDK Pho85 with its cyclin Pcl1 [77,96]. The Efg1 and Cph1 transcription factors activate various genes, but a central target is the transcription factor gene *UME6* [85,90]. Ume6 functions in a large measure via the induction of a hyphal-specific cyclin gene, *HGC1* [97]. Hgc1, a cyclin of the cell-cycle CDK Cdc28 that is essential for hyphal morphogenesis, is a homolog of the *S. cerevisiae* G1 cyclins Cln1 and Cln2 [29]. Hgc1 in conjunction with Cdc28 phosphorylates several effectors of morphogenesis, including the Cdc42 GAP Rga2, a central regulator of polar growth [98]; the GEF Sec2, a regulator of polarized secretion at the tip of the hyphae [99]; the exocyst component Exo84 [100]; the septin Cdc11 [101]; and the Spa2 polarisome scaffold protein, maintaining it at the hyphal tip [102]. A simplified hyphal induction pathway, activated by a single stimulus—low nitrogen—is depicted in Figure 1. 

**Figure 2:**
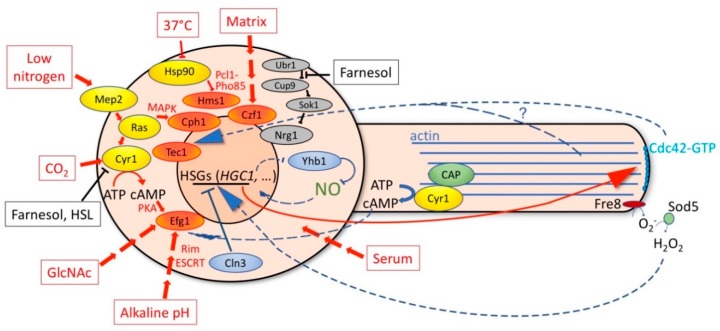
Schematic depiction of some of the positive stimuli on hyphal induction (boxed in red and red arrows) and inhibitory signals (black). The cellular mediators of the hyphal morphogenetic pathways are depicted in yellow ovals and the transcription activators that induce hyphal growth are in orange ovals. The internal signals feeding back from the morphogenetic apparatus (dashed lines) and the cell cycle apparatus (solid lines) to the hypha-specific gene (HSG) expression program are shown in blue. See text for details of the individual interactions. The question mark indicates that it is still unclear whether Cdc42 affects the HSG expression directly or indirectly via its effect on the cytoskeleton.

The pathway shown in Figure 1 is linear, leading from nitrogen starvation via cellular signal transduction pathways to the activation of transcription factors, which in turn leads to the expression of direct effectors of hyphal morphogenesis. This scheme is highly simplified: for example, the Mep2 receptor is involved in nitrogen starvation sensing [91], while other stimuli such as alkaline pH or GlcNAc, activate other receptors [103,104]; *HGC1* is transcribed by Ume6 during hyphal extension, but initial activation relies on other TFs [97]. More comprehensive descriptions of the various pathways involved in hyphal induction under different conditions and by alternative stimuli can be found in recent reviews [22,105]. In common with the scheme in Figure 1, these parallel pathways (examples of which are shown in red in Figure 2 below) all involve linear transduction of signals from the environment via gene expression regulation to the activation of hyphal effector proteins. These signals also include quorum sensing factors [106], of which the best studied is farnesol [107], a molecule that inhibits hyphal morphogenesis by affecting many hyphal-inducing pathways via the direct inactivation of Cyr1 [108], via the indirect stabilization of the transcriptional repressor Nrg1 [109], and via an unknown mechanism involving the Eed1 protein [110]. Thus, the individual fungal cell integrates the inputs of many external stimuli in order to determine whether to initiate hyphal morphogenesis. However additional internal cellular signals also feed into the hypha-specific gene (HSG) expression mechanism. These are discussed below.

While many genes were shown to be involved in hyphal development under several or under specific conditions, only some of these can induce hyphal development in the absence of external stimuli when ectopically expressed. These include the genes for Ras1^G13V^ [56,111,112], Ste11ΔN [113], Ume6 [90] and Hgc1 [113], which can all induce the switch from yeast to hyphae and hyphal-specific gene expression under yeast growth conditions, particularly when expressed in the activated form.

A large number of genes are typically induced following the stimulation of hyphal growth from a yeast culture (the hypha-specific genes (HSG)) [69,70]. However, only a few of these genes are actually essential for hyphal morphogenesis. Chief among them are the *UME6* and *HGC1* genes, described above. Conversely, according to one report at least, it appears that under some circumstances, hyphal morphogenesis can be induced without any detectable induction of HSGs [114]. Furthermore, some HSGs such as *RBT5* can also be expressed in yeast-form cells under certain conditions such as iron starvation [115,116]. Thus, it was suggested that the hypha-specific genes (HSGs) might be more correctly referred to as hypha-associated genes (HAGs) [105]. Nonetheless, we will retain the customary nomenclature here and call these genes HSGs. 

## 5. Regulation of Hyphal Morphogenesis and HSG Expression by Internal Signals

### 5.1. Interference with Yeast Proliferation Induces Hyphal Morphogenesis and HSG Expression

The established role of the *S. cerevisiae* cell-cycle CDK in the coordination of cell cycle progression with bud morphogenesis [43], as well as its role in *S. cerevisiae* pseudohyphal growth [117,118], led to the exploration of the role of cell cycle regulators in *C. albicans* morphogenesis [119]. Interference with cell cycle progression in the absence of external stimuli was found to induce hyphal-like growth; this hyphal morphogenesis is often accompanied by an induction of HSG expression. The types of cell cycle interferences that induce hyphal growth and HSG expression include mitotic inhibition by the depletion of the essential mitotic polo kinase Cdc5 [120,121], S-phase inhibition by the addition of the DNA synthesis inhibitor hydroxyurea, or through the depletion of the ribonucleotide reductase Rnr1, other genotoxic stresses [121,122], or cell cycle arrest in G1 through the depletion of the essential G1 cyclin Cln3 [123,124]. Interference with the activity of the SCF ubiquitin ligase complex, which in *S. cerevisiae* is essential for the G1-to-S transition [125], by mutating its essential cullin component Cdc53 or by interfering with its Rub1 modification, also induced a filamentous morphology, albeit pseudohyphal rather than hyphal in most cases [126,127]. In contrast, the deletion of *CDC4*, which encodes one of the alternative substrate recognition components of the SCF complex, led to a strong constitutive hyphal phenotype with aerial hyphae protruding from colonies [128]. The constitutive hyphal phenotype of the *cdc4^−/−^* mutant is a consequence of the combined stabilization of two SCF^CDC4^ substrates, the CDK inhibitor Sol1, which delays cell cycle progression at the G1-to-S transition, and the TF Ume6, which promotes hyphal extension [129].

Diminished expression of the essential cell cycle kinases Cak1, an activator of the cell cycle CDK Cdk1/Cdc28, or of Kin28, involved in RNA polymerase II activity, also causes filamentous growth and HSG expression, and so do the reduced activity of mutants in a number of additional essential genes, a phenomenon that has been called "essential process impairment-induced filamentation" [130,131]. Conversely, the acceleration of the cell cycle either by deletion of the cell cycle inhibitor Nrm1 [132] or by the overexpression of Cln3 [129] inhibits hyphal morphogenesis and HSG expression. The pathways for hyphal induction upon interference with the cell cycle progression include the transcription factor Ume6 and the Cdc28 cyclin Hgc1 in the case of the depletion of Cdc5 or Cln3, but not for hydroxyurea-induced filamentous growth. The latter, however, still required the Ras-cAMP pathway activity [133,134]. HSG expression induced by diminished *CAK1* expression was surprisingly resistant to the deletion of TFs that are normally essential for hyphal growth under many conditions, in particular, Ume6 and Brg1. Deletion of both *UME6* and *BRG1* together did, nonetheless, greatly reduce HSG expression in the diminished *CAK1* expression background, suggesting that several parallel HSG expression pathways are induced in this background [130]. The signals that induce hyphal morphogenesis upon cell cycle inhibition or essential process impairment, in general, are yet unknown. 

### 5.2. Actin Depolymerization Blocks Hyphal Morphogenesis and Inhibits HSG Expression

The linear pathway shown in Figure 1 would suggest that after induction of hyphal morphogenesis by external stimuli, interference with the actin cytoskeleton using actin depolymerizing drugs such as cytochalasin A and latrunculin A should inhibit cellular morphogenesis, but not HSG expression. This is, however, not what was observed. These two drugs inhibited hyphal morphogenesis, but also caused a strong suppression of HSG expression [58,135]. Similar suppression was noted with mutants that are defective in actin nucleation but, conversely, the drug jasplakinolide that inhibits hyphal elongation by stabilizing filamentous actin was not defective in HSG expression [135]. These observations suggest the existence of a mechanism linking the filamentous actin cytoskeleton, possibly via cAMP levels [135], to the regulation of HSG expression. This mechanism may involve the adenylyl cyclase Cyr1, which is essential for the hyphal morphogenesis gene expression program and which, via the Cap1 protein, binds to actin [136]. However, the details of this mechanism are unknown. 

### 5.3. Inhibition of Endocytosis Inhibits Hyphal Morphogenesis and HSG Expression

As mentioned in Section 2, hyphal morphogenesis requires that exocytosis be balanced by endocytosis, and indeed, a collar of endocytic actin patches is located sub-apically in growing hyphae. Thus, not surprisingly, when general endocytosis is inhibited either pharmacologically [113] using Trifluoperazine or related compounds, or genetically through the deletion of the BAR proteins Rvs161 or Rvs167 [137], or by overexpressing the inhibitory Akl1 kinase [138], hyphal extension is inhibited. However, in addition, HSG expression is also strongly inhibited under these conditions [113,138]. Furthermore, the activation of endocytosis through deletion of the inhibitory kinase Akl1 or through the overexpression of the endocytic scaffold protein Pan1 both increased the rate of hyphal elongation and the expression of HSGs [138]. Since there is no known connection between the endocytic proteins Akl1 and Pan1 and the signal transduction pathways of hyphal induction, it appears that interference with the mechanism of hyphal extension can indirectly affect the HSG expression.

## 6. Feedback Regulations in Hyphal Morphogenesis

### 6.1. Positive Feedback Regulations of Hyphal Transcription

To achieve sustained hyphal extension may require positive feedback (or "feed-forward") regulations in order to ensure the continuation of the HSG expression program beyond its initial activation. One instance of a feed-forward regulation is that of the transcription factor Ume6, whose gene is activated under hyphal induction conditions. Ume6 was shown to activate its own gene’s promoter under hyphal induction conditions and this reinforcing feedback loop was essential for the maintenance of hyphal elongation [139]. Another mechanism that could contribute to the persistence of the HSG expression program relies on chromatin modifiers that perpetuate an active chromatin state by histone deacetylation. The initiation of HSG expression induces the down-regulation of the expression of a transcriptional repressor, Nrg1 [140], as well as the degradation of the Nrg1 protein [109], causing *inter alia* the activation of the TF gene *BRG1* [87]. Brg1 then recruits the deacetylase Hda1 to HSG promoters, which causes histone deacetylation indirectly through the inactivation of the NuA4 histone acetyltransferase complex [87,141]. Upon Nrg1 removal, Brg1 can itself also activate its own promoter in another feed-forward loop that can perpetuate the expression of HSGs [141]. 

### 6.2. Cdc42

The small GTPase Cdc42 is an essential protein for yeast growth and proliferation and it is also specifically involved in hyphal elongation. Misregulation of Cdc42 activity or localization either through mutation, by increasing or decreasing its expression, or by manipulating its associated GTP exchange factor (Cdc24) or its associated proteins Bem1 and Rsr1, causes a disruption in hyphal morphogenesis [58,59,61,63,142,143]. Interestingly, in the absence of proper hyphal morphogenetic Cdc42 activity, including properly focused localization at the hyphal tip, the initial HSG expression cannot be sustained [61,63,143]. This could be due to the role of the Cdc42 activity in actin cytoskeleton polarization which, in turn, can affect the HSG expression program, as discussed above. On the other hand, the toxicity of the activated *CDC42*^G12V^ allele could be suppressed by the deletion of the MAP kinase signaling pathway protein Cst20, suggesting that in addition to its cytoskeletal role in hyphal morphogenesis, Cdc42 might be directly involved in the transduction of the hyphal induction signals via the MAPK pathway [59]. Nonetheless, additional observations indicate that Cdc42 plays a role in maintenance, rather than in the initial induction, of the HSG expression during hyphal morphogenesis [61]. Furthermore, the focused localization of the Cdc42 protein at the hyphal apex is necessary for its effect on HSG expression [63]. Thus, whereas a direct role for Cdc42 in the MAPK signal transduction pathway cannot be excluded, it is likely that the Cdc42 morphogenetic complex impacts HSG expression indirectly via its effect on hyphal morphogenesis. 

### 6.3. Reactive Oxygen and Nitrogen Species

Nitric oxide (NO) and reactive oxygen species (ROS) such as the superoxide radical and hydrogen peroxide are toxic compounds generated by host phagocytic cells in response to invading microorganisms. Conversely, the microorganisms have evolved mechanisms to detoxify these reactive species (recently reviewed in Reference [144]). In addition, exogenous H_2_O_2_ was shown to promote, at sub-toxic levels, polarized cellular growth [145]. Interestingly, however, a recent report indicates that the *C. albicans* hyphal cells can themselves generate H_2_O_2_ via disproportionation through the superoxide dismutase Sod5 of the superoxide ion generated by the NADPH oxidase Fre8 at the hyphal tip, and that this H_2_O_2_ contributes to hyphal morphogenesis [146]. *FRE8* is itself strongly induced under hyphal growth conditions, potentially providing a reinforcing loop that sustains morphogenesis using this "autocrine" mechanism. 

In another recent study, endogenous NO was found to be associated with hyphal morphogenesis: the pharmacological inhibition of NO generation prevented hyphal morphogenesis via the prevention of the normal degradation of the HSG suppressor Nrg1 under hyphal induction conditions [147]. Consistent with the notion that elevated NO promotes hyphal morphogenesis, the deletion of the main NO detoxifying enzyme, Yhb1, caused hyper-filamentation [148]. *YHB1* expression is suppressed under hyphal induction conditions [149], providing another potential reinforcing feedback regulation on hyphal morphogenesis.

## 7. Conclusions

The hyphal development program, once initiated, likely requires commitment mechanisms in order to be sustained. Commitment could be achieved by positive feedback regulatory loops such as the transcriptional feed-forward loops, the effects of actin cytoskeleton polarization on the HSG expression program, and the ROS- and NO-mediated regulation of hyphal morphogenesis described above. The internal signals that can affect hyphal gene expression are schematically indicated in Figure 2 with blue arrows. 

Besides the external hyphae-inducing stimuli that are transmitted via well-studied transduction pathways to the nucleus to induce the HSG expression program and hyphal growth (red arrows in Figure 2), a significant number of cellular conditions have been identified that can also induce hyphal morphogenesis. These include mutations or treatments that inhibit cell cycle progression or otherwise inhibit essential cellular processes. Given that the abilities to form hyphae and to generate biofilms are correlated, it was suggested that hyphal induction upon the inhibition of proliferation represents an evolved cellular response, which maximizes cell attachment and substrate penetration upon encountering adverse conditions, thereby enabling, e.g., biofilm formation as a coping mechanism [131]. This possibility implies the existence of sensors of cellular growth capacity that transmit information on the internal state of the cell to the HSG expression system. One candidate for such a sensor is the essential G1 cyclin Cln3: high levels of Cln3 promote yeast proliferation and inhibit hyphal morphogenesis [129,138], whereas reduced Cln3 levels promote hyphal morphogenesis [123,124,130]. It is not known how *C. albicans* Cln3 levels are regulated, but one possibility is that by analogy with *S. cerevisiae*, where Cln3 protein levels are strongly associated with cell proliferation rates [150], the *C. albicans* Cln3 cyclin likewise serves as a sensor of the cellular biosynthetic capacity.

It is, however, also possible that rather than being an evolved function, the cell cycle inhibition-mediated hyphae formation is a consequence of mechanisms that normally ensure commitment to hyphal morphogenesis. For example, cell cycle inhibition could induce an initial cytoskeletal polarization due to, e.g., an imbalance between the actions of the different Cdc28 cyclins. This initial polarization could then be further amplified to the extent that cytoskeletal polarization can affect the HSG expression, leading to hyphal morphogenesis in the absence of regular stimuli. The cell cycle inhibition-induced mode of hyphal morphogenesis could thus represent a spurious consequence of the existence of the positively reinforcing cellular mechanisms that ensure commitment to morphogenesis after germ tube initiation. 

The possibilities that cell cycle inhibition-induced hyphal morphogenesis represents an evolved response or that it is a consequence of reinforcing feedback loops in the morphogenetic pathway that are activated under certain artificial conditions are not mutually exclusive. In either case, future challenges will include the elucidation of the molecular mechanisms that connect the hyphal extension apparatus to the HSG expression program. Cell cycle inhibition-induced hyphal morphogenesis, regardless of its evolutionary basis, provides a useful experimental window into the mechanisms that sustain hyphal growth.
